# Toward scalable species descriptions for dark taxa: the role of molecular data

**DOI:** 10.3897/zookeys.1283.172693

**Published:** 2026-06-30

**Authors:** Michael J. Sharkey, Marko Mutanen, Austin Baker, Brian V. Brown, Buntika Areekul Butcher, Ashley P. G. Dowling, H. Charles J. Godfray, Winnie Hallwachs, Axel Hausmann, Paul D. N. Hebert, Diego J. Inclán, Daniel H. Janzen, Niina Kiljunen, Scott E. Miller, Guilherme Oliveira, Laurence Packer, Donald L. J. Quicke, Jeffrey H. Skevington, Dirk Steinke, José A. Hernández Ugalde, Alfried P. Vogler, Emily Hartop

**Affiliations:** 1 The Hymenoptera Institute, 1100 Pepperhill Circle, Lexington, Kentucky 40502, USA Department of Biology, University of Pennsylvania Philadelphia United States of America; 2 Ecology and Genetics Research Unit, University of Oulu, PO Box 8000, FI-90014, Oulu, Finland National Museum of Natural History, Smithsonian Institution Washington, DC United States of America; 3 Entomology Section, Natural History Museum of Los Angeles County, 900 Exposition Blvd, Los Angeles, CA 90007, USA Entomology Section, Natural History Museum of Los Angeles County Los Angeles United States of America; 4 Center of Excellence in Integrative Insect Ecology, Department of Biology, Faculty of Science, Chulalongkorn University, Phayathai Road, Pathumwan, BKK 10330, Thailand Facultad de Ciencias Agrícolas, Universidad Central del Ecuador Quito Ecuador; 5 Department of Entomology & Plant Pathology, University of Arkansas, Fayetteville, Arkansas, 72701, USA Center of Excellence in Integrative Insect Ecology, Department of Biology, Faculty of Science, Chulalongkorn University Pathumwan Thailand; 6 Department of Biology, Oxford University, South Parks Rd., Oxford, OX1 3PS, UK Instituto Nacional de Biodiversidad Quito Ecuador; 7 Department of Biology, University of Pennsylvania, Philadelphia, PA, USA Department of Life Sciences, Natural History Museum London United Kingdom; 8 SNSB - Bavarian State Collection of Zoology, Münchhausenstr. 21, 81247 Munich, Germany Ecology and Genetics Research Unit, University of Oulu Oulu Finland; 9 Centre for Biodiversity Genomics, University of Guelph, Guelph, Ontario, N1G2W1, Canada Department of Life Sciences, Imperial College London Ascot United Kingdom; 10 Instituto Nacional de Biodiversidad, Quito, Ecuador SNSB - Bavarian State Collection of Zoology Munich Germany; 11 Facultad de Ciencias Agrícolas, Universidad Central del Ecuador, Quito, Ecuador Centre for Biodiversity Genomics, University of Guelph Guelph Canada; 12 National Museum of Natural History, Smithsonian Institution, PO Box 37012, MRC 105 Washington, DC 20013-7012, USA Department of Biology, Oxford University Oxford United Kingdom; 13 Instituto Tecnológico Vale, Rua Boaventura da Silva 955, Belém, PA, 66055-090, Brazil Department of Biology, York University Toronto Canada; 14 Department of Biology, York University, 4700 Keele Street, Toronto, Ontario M3J1P3, Canada Instituto Tecnológico Vale Belém Brazil; 15 146 Monty Drive, Ottawa, ON, K0A3M0, Canada Department of Natural History, University Museum, Norwegian University of Science and Technology Trondheim Norway; 16 Technical Area, National Commission for Biodiversity Management, C. 9 y 11Avenida 21, San José, Costa Rica The Hymenoptera Institute Lexington United States of America; 17 Department of Life Sciences, Natural History Museum, London, SW7 5BD, UK Department of Entomology & Plant Pathology, University of Arkansas Fayetteville United States of America; 18 Department of Life Sciences, Imperial College London, Silwood Park Campus, Ascot SL5 7PY, UK Unaffiliated Ottawa Canada; 19 Department of Natural History, University Museum, Norwegian University of Science and Technology, 7012 Trondheim, Norway Technical Area, National Commission for Biodiversity Management San José Costa Rica

**Keywords:** COI, DNA barcode, integrative taxonomy, taxonomic impediment

## Abstract

Species descriptions remain the foundation of biodiversity science, but alpha taxonomy faces a twofold challenge: accelerating the pace of species discovery while ensuring that descriptions produce data that are usable, comparable, and reproducible. This is particularly acute for “dark taxa”—small, hyperdiverse, and morphologically challenging groups—for which morphology-based workflows alone often fail to deliver scalable and reliable identifications. As a result, most species remain undescribed, and many described species are difficult or impossible to identify.

We argue that species descriptions must be integrative in a way that meets the practical requirements of modern taxonomy, including scalability, reliable identification, reproducibility, and applicability across life stages. At present, standardized molecular data in the form of DNA barcoding is the only approach that consistently satisfies these criteria. Emerging approaches based on robotics, imaging and artificial intelligence may eventually provide complementary frameworks, but currently lack the standardization and interoperability required for routine application at scale. In contrast, barcode data already enable high-throughput species delimitation, straightforward identification, and direct comparability across studies, supported by global reference libraries and standardized protocols.

We therefore propose that DNA barcodes be treated as a baseline component of species descriptions for invertebrates. Standardizing molecular data in taxonomy will accelerate species discovery, improve reproducibility and stability, and ensure that newly described taxa remain accessible in an increasingly DNA-based research landscape.

## Introduction

For more than two centuries, species descriptions have been grounded primarily in morphological traits, with ecological data—such as diet, habitat, phenology, and mating signals—added only sporadically. While this approach has yielded a rich legacy of taxonomic knowledge, it is increasingly strained by two fundamental challenges: the need to accelerate the pace of species discovery, and the need for species descriptions to generate data that are broadly usable, comparable, and reproducible.

These challenges are especially acute for “dark taxa”—species-rich lineages composed of small, inconspicuous organisms that remain undescribed and difficult to delimit using morphology alone. Originally coined to describe unidentified entries in GenBank (Page 2011), the term has evolved to encompass hyperdiverse (>1000 species predicted) taxa for which the absence of formal species descriptions is particularly acute (10% or less are known) (Hausmann et al. 2020; Hartop et al. 2022). In such groups, traditional workflows are too slow and often fail to produce descriptions that support reliable identifications, resulting in large backlogs of undescribed diversity and limited accessibility of existing taxonomic knowledge.

Despite this, and despite long-standing recognition of the limitations of morphology for identification (Packer et al. 2009), taxonomic treatments solely reliant on morphology remain common. For example, in two recent standard issues (1221 + 1223) of ZooKeys, 15 of the 26 papers (~58%) in which new species were described lacked molecular data. Similarly, in Zootaxa, of the first 30 open access papers describing new arthropod species in 2025, only 13 (43%) used DNA data. Even more concerning is the fact that among 30 recent papers describing new mite species, a definitive dark taxon, 28 (93.3%) employed morphology only. This stands in stark contrast to recent progress in other disciplines. Not long ago, mycology faced a similar challenge – but by 2018, 94% of mycological descriptions included molecular data, while at the same time sequence data were absent from 85% of entomological publications (Miralles et al. 2020).

The emergence of inexpensive, high-throughput DNA sequencing methods (Hebert et al. 2018, 2025; Srivathsan et al. 2021) has presented a transformative opportunity to address these constraints. We argue that it is both timely and necessary for taxonomists—especially those working on dark taxa—to incorporate molecular data as a standard component of species descriptions.

Here, we present twelve reasons why incorporating standardized molecular data, specifically DNA barcoding, is essential for meeting these criteria. Although these arguments are most immediately applicable to dark taxa, they extend to more well-characterized (“illuminated”) groups as well. Importantly, we do not advocate for “molecular-only” taxonomy; rather, we argue that molecular data currently provide the only framework capable of fulfilling these requirements in a consistent and scalable way, while complementing and strengthening morphology. Emerging approaches may eventually provide alternative data streams that meet these criteria, but such systems are not yet operational for alpha taxonomy at scale in standardized and widely accessible ways (see discussion).

1. **Scale** — Molecular approaches can treat thousands of species at a time.
2. **Identification simplicity** — Identification using sequence data is relatively straightforward.
3. **Misleading matches** — Molecular data prevent false identifications based on incomplete keys.
4. **Universality** — Sequence data are universally interpretable and digitally shareable.
5. **Developmental inclusivity** — DNA can identify any sex or life stage.
6. **Comprehensive coverage** — Molecular surveys can include undescribed diversity.
7. **Cryptic diversity** — Molecular tools can resolve morphologically cryptic species.
8. **Less dependence on physical access to type specimens** — Sequence data lessen reliance on physical access to types.
9. **Community expansion** — DNA barcoding enables reliable identification by non-specialists.
10. **Reproducibility** — Molecular data enable reproducible and quantitative species boundaries.
11. **Taxonomic stability** — Sequence-based frameworks reduce synonymy and misidentification.
12. **Future relevance** — DNA-based identification underpins emerging approaches (e.g., environmental DNA, metabarcoding), and taxa lacking such data risk exclusion.

## Results

Our aim is not to advocate for the replacement of morphology or other sources of evidence, but to evaluate how integrative taxonomy (Dayrat 2005; Padial et al. 2010) can be implemented in a way that satisfies the practical requirements of scalable, reproducible, and broadly accessible taxonomy. We treat integrative taxonomy as the goal and find that DNA barcodes currently provide the only data type that consistently meets these requirements. We therefore argue that they should form a standardized component of new species descriptions.

We acknowledge that molecular data are not a universal solution. DNA barcodes do not resolve all species boundaries, and access to sequencing infrastructure and expertise remains uneven across research communities. However, these challenges do not diminish the value of molecular approaches; rather, they highlight the importance of their thoughtful application. In line with best practices in integrative taxonomy, molecular data should be interpreted alongside other relevant sources of evidence, including morphology, ecology, and behavior. At the same time, rapid advances in sequencing technologies are making molecular methods increasingly affordable, portable, and accessible, lowering barriers to adoption and enabling broader participation in taxonomic research.

The following sections examine the twelve key criteria, with examples drawn from recent taxonomic publications. Across these criteria, we show that standardized DNA barcode data uniquely fulfill these requirements in a consistent and operational way, while other data sources remain complementary but insufficient on their own.

### 1. Scale

For truly dark taxa such as Ichneumonoidea (Hymenoptera), small flies (Cecidomyiidae, Ceratopogonidae, Phoridae), nematodes, and mites, none of the published approaches, including the minimalist methods of Meierotto and Sharkey (Meierotto et al. 2019; Sharkey et al. 2021, 2023) will solve the taxonomic impediment. Sharkey explored the possibility of employing the minimalist method to describe all Costa Rican species of Braconidae. However, at a rate of 1,000 species per year it would take more than 40 years to complete this task, which exceeds his realistic lifespan. How many taxonomists are there, or will there be, prepared to dedicate their lives to describing the braconid species of Colombia, Brazil, China, etc.? Documenting biological diversity is undeniably urgent (Löbl et al. 2023). Several strategies have been proposed to address the challenge of scale in taxonomy. These include prioritizing subsets of biodiversity (e.g., economically important or threatened taxa), adopting minimalist or rapid-description approaches, and developing new technologies such as automated imaging, machine learning, and high-throughput morphological analysis. While each of these approaches offers potential benefits, they do not currently provide a generalizable solution for hyperdiverse dark taxa. Prioritization necessarily leaves large portions of biodiversity undescribed, while morphology-based acceleration remains constrained by the need for expert interpretation and comparison with existing material. Emerging technological approaches show promise but are not yet sufficiently standardized, scalable, or widely implemented to address the magnitude of the taxonomic impediment.

To illustrate the immensity of the problem, consider that there are ~123 species of scuttle flies (Diptera: Phoridae) described from Canada (Savage et al. 2019). It has taken more than a century to assemble these, but there are presently (24 April 2025) 1,095 Canadian BINs (putative species) (Ratnasingham and Hebert 2013) of Phoridae on the Barcode of Life Data System (BOLD). This means that less than 11% of the barcoded Canadian phorids are described and the family is far from being completely collected. For Costa Rica, which is not as well surveyed as Canada, the percentage of undescribed Phoridae is even greater. There are 8,057 BINs of Costa Rican phorids on BOLD (24 April 2025) and only ~400 phorids are described from this nation (online phorid catalog www.phorid.net/pcat, accessed 29 May 2025). Even more stunning is that there are an estimated 2,112 species of Phoridae from a single site in Costa Rica, with projected number of >3,000 (Brian Brown, unpublished). This estimate approaches the number of described world species, and is almost surely an underestimate of the site, since only ground-level Malaise traps were used. Similarly, in the case of Canadian mites there are 2,999 described species while BOLD has 7,694 BINs (24 April 2025). Remarkably, this latter count is based on specimens that were primarily captured by Malaise traps, a supremely inefficient collection method for the group.

Molecular approaches provide one of the few realistic pathways for scaling taxonomy to match the magnitude of Earth’s undocumented biodiversity. DNA barcoding enables the rapid generation of species hypotheses from thousands of specimens, which can subsequently be tested using morphology and other complementary data sources within an integrative framework (Hartop et al. 2022). The need for such scalability is particularly acute in hyperdiverse groups. Sharkey et al. (2021), for example, described 120 species of Costa Rican *Chelonus* and *Bracon* using barcode-informed workflows, yet 596 BINs are now known from these genera in Costa Rica alone (BOLD accessed 17 June 2026), suggesting that much of their diversity remains undocumented. This gap between known and estimated diversity is not unique: Meierotto et al. (2019) estimated that, at current rates of species description, documenting the world’s Ichneumonoidea would require between 535 and 1,817 years. Such figures illustrate why scalable, molecularly informed approaches are increasingly essential for addressing the taxonomic impediment.

Critically, molecular data do not eliminate the need for morphology or other forms of data, but they currently provide the only broadly applicable and operational framework for scaling species discovery across hyperdiverse taxa. Complementary data sources can be used to validate, and iteratively enrich species descriptions, whereas attempting to scale taxonomy without a molecular backbone is unlikely to succeed.

### 2. Identification simplicity

In dark taxa, the sheer number of species makes reliable identification using morphology alone increasingly impractical. Morphological keys often become extremely long and complex, with hundreds or thousands of couplets. In practice, experienced taxonomists rarely use such keys in a strictly linear fashion, instead relying on a combination of illustrations, selective couplets, and prior knowledge. However, this approach depends heavily on expertise and does not scale well to non-specialists or to the identification of large numbers of specimens.

**Figure 1.**
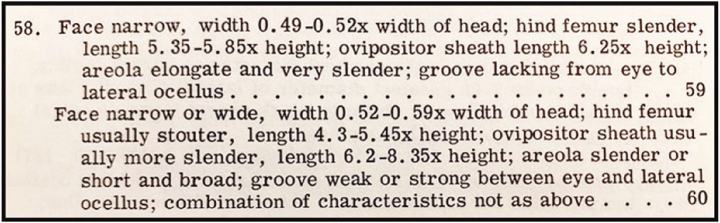
Couplet 58 in the key to Neotropical *Mesochorus* by Dasch (1974) illustrating the difficulty of morphological identification due to character state overlap, features that are indecisive (“usually”), and the need for accurate measurements (slender versus very slender).

A clear example is the key to Neotropical hyperparasitoid wasps of the genus *Mesochorus* Gravenhorst (Hymenoptera: Ichneumonidae) (Dasch 1974). It includes 277 species ranging from Mexico to southern South America. When treating 158 Costa Rican species, Sharkey et al. (2023) found the key effectively unusable. At 279 couplets long, it relies heavily on subjective judgements, such as those required in the couplet illustrated in Fig. 1. The key also incorporates fine measurements with overlapping values. Imprecise statements like “usually” fail to identify some proportion of specimens, and qualitative descriptors such as “elongate” and “slender” are, even with illustration, ambiguous at best. These comments are not a criticism of Dasch but of the framework within which taxonomists worked at the time.

The problem scales rapidly with increasing diversity. Sharkey et al. (2023) estimated that there are ~688 species of *Mesochorus* in Costa Rica alone. If this represents just 10% of the Neotropical fauna (the ratio for well-studied groups, e.g., tree species, Cazzolla Gatti et al. 2022), there are likely to be ~6,880 Neotropical species of *Mesochorus*. Their identification would require a dichotomous key with almost 7,000 couplets, rendering it effectively unusable for routine or large-scale identification. Similar problems were encountered in Vikberg and Vårdal’s (2017) attempt to identify Swedish specimens of *Mesochorus*, even with a recent key to the European fauna (Schwenke 1999). “The specimens could not be identified using existing keys to European species …, therefore a study of museum specimens (including primary types) was initiated.” (Vikberg and Vårdal 2017: 4). It is not only the length of keys that are problematic. Even at the generic level, keys permitting the identification of new insect genera were provided in fewer than one-third of more than 420 papers reviewed by Packer (2024), and on average these keys did not meet one-third of 17 optimality criteria that he suggested. In theory, tabular or multi-access digital keys offer a more flexible alternative to traditional dichotomous keys and are often used in combination with images and expert knowledge. However, in practice, they suffer from the same fundamental limitations: too many species, too much character overlap, missing data, and too many undescribed taxa to support reliable identifications.

As an—admittedly imperfect—response to these limitations, Sharkey et al. (2023) diagnosed the species of Costa Rican *Mesochorus* using DNA barcodes supplemented with images and host data. More broadly, this reflects a shift toward identification frameworks that reduce reliance on subjective morphological interpretation. Matching query sequences to reference libraries enables rapid and repeatable identification with minimal reliance on expert interpretation. Although morphology and image-based methods remain valuable complements, molecular identification currently represents the most broadly applicable approach for large-scale identification of dark taxa.

### 3. Misleading matches

Morphological keys can be highly effective for well-known taxa in well-studied and geographically constrained regions. However, in dark taxa, they frequently produce misleading identifications. As noted by Packer et al. (2009) and others, “Keys are written by those who don’t need them for those who can’t use them.”

A clear example involves the Neotropical braconid wasp genus *Alabagrus* Enderlein. Yamada et al. (2006) used Sharkey’s (1988) morphological key to identify 21 species of *Alabagrus* from Brazil including ten taxa whose holotypes originate from Mexico, Central America, and the United States. The likelihood of these species occurring in Brazil is extremely low, yet they presumably fit the key. We estimate there to be more than 1,000 species of *Alabagrus* in the Americas (MS, unpublished) and only ~10% of them have been described (Sharkey et al. 2018). Many of the 90% of undescribed species will run comfortably somewhere in the key resulting in an incorrect identification. Of the six barcoded species of *Alabagrus* from the United States and Canada, none has been found in Costa Rica. There are only nine South American species on BOLD (19 Aug 2025), all from Peru, and none of these is known outside that country. The species identified incorrectly by Yamada et al. (2006) are likely endemic to Brazil, or that broad region, and as a result many erroneous distributional species records for *Alabagrus* are in the literature.

A similar example comes from the genus *Chelonus* (Braconidae). Sandoval-B et al. (2024) recorded seven species of this genus from the Galapagos Islands. According to their methods section, “Specimens were identified to species using revisionary taxonomic keys provided by Papp (2010, 2016)”, suggesting that holotypes were not examined. The keys resulted in the following identifications with the type locality of the holotype in parentheses: *Chelonus buscki* Viereck (Trinidad); *Chelonus carinatus* Provancher (Canada); *Chelonus johni* Marsh (Colombia); *Chelonus refluus* (Papp) (Honduras); *Chelonus sulcifera* (Papp) (Costa Rica); *Chelonus topali* (Papp) (Argentina); and *Chelonus turgoclarus* (Papp) (Ecuador). There are clues suggesting that these identifications are weakly supported and, in some cases, almost certainly wrong. The most obvious is the identification of *C. carinatus* because its holotype is from Canada. The probability that a Canadian species of *Chelonus* thrives in the tropical Galapagos Islands is remote. BOLD contains 93 BINs of *Chelonus* from Canada and 171 BINs from Costa Rica. None of the Canadian BINs is found in Costa Rica, or anywhere else in the Neotropics so there is no reason to believe that *C. carinatus* is a synanthropic species.

Because most New World species of *Chelonus* are undescribed, the probability that all seven species known from the Galapagos are described is extremely low. Presently (18 Aug 2025) there are 196 BINs of *Chelonus* in Costa Rica and there are likely to be at least twice this number that await collection and barcoding. To get a rough idea of how many species of *Chelonus* there are in the Neotropics we can extrapolate from the number of tree species in Costa Rica compared to the remainder of the Americas. The number of tree species in Area de Conservación Guanacaste was estimated by Nelson Zamora (Zamora et al. 2000–2004) to represent ~ 10% of all New World tree species (Cazzolla Gatti et al. 2022). If Costa Rican *Chelonus* follow this same pattern it suggests the presence of 3,420 species of *Chelonus* in the Americas, but only 136 species have been described from the Neotropics (Papp 2010, 2016; Sharkey et al. 2021). Coupling these estimates with the assumption that any Neotropical species, described or undescribed, has an equal chance of occurring on the Galapagos, the probability of even one species from the Galapagos being a previously named species is 136/3,420 = ~0.04 (4%). Following the same reasoning, the probability that a species from the Galapagos is undescribed is ~0.96 (96%). The probability that all seven species on the Galapagos are described species, as proposed in Sandoval-B et al. (2024), is vanishingly small (0.04^7^ = 1.6 × 10^-7^). Aside from the species with the Canadian holotype, *C. carinatus*, five of the other six holotypes are from distant localities (e.g., Argentina and Trinidad) and are not likely to have distributions that extend to the Galapagos Islands. Without molecular data, the seven species records for the Galapagos Islands will be exceedingly difficult to refute or corroborate.

It is important to note that DNA barcode reference libraries are also incomplete and currently represent only a fraction of global biodiversity. As a result, many query sequences (especially for dark taxa) will not match a named species. However, the consequences of this incompleteness differ fundamentally from those associated with morphology-based identification. In morphological frameworks, unknown species are often forced into existing keys, resulting in confident but incorrect identifications. In contrast, molecular approaches allow specimens to remain unassigned or to be recognized explicitly as distinct, unnamed lineages (e.g., clusters or BINs), thereby avoiding the propagation of erroneous species names. Even in the absence of exact matches, query sequences can be placed within clusters of closely related taxa, allowing specimens to be compared with their nearest relatives and providing a meaningful framework for further study.

These examples illustrate a general problem: in hyperdiverse groups with incomplete taxonomic coverage, morphological keys do not simply fail, they can actively mislead by forcing unknown species into existing frameworks. Alternative approaches, such as digital or multi-access keys, do not resolve this issue, as they depend on the same incomplete and overlapping character data.

In contrast, DNA barcode-based identification reduces the risk of misleading matches by enabling direct comparison of specimens through sequence similarity. When no close match exists, specimens can be recognized as distinct lineages rather than being forced into incorrect species assignments, with unmatched sequences explicitly signaling undescribed diversity. Although not infallible, this approach provides a more transparent and testable basis for identification than morphology-based systems that lead to assignment within incomplete frameworks. In this sense, molecular approaches make uncertainty explicit, whereas morphology-based identification often obscures it. As barcode reference libraries continue to expand, the accuracy and resolution of these assignments will improve.

### 4. Universality

Morphological terminology is highly specialized and often accessible only to a small group of experts. For example, keys to Costa Rican species of *Alabagrus* (Sharkey et al. 2018) employ terminology (e.g., mesoscutum, gena, propodeum, precoxal sulcus, foveolate, metapleuron, frons, vertex) familiar primarily to specialists in Hymenoptera. Even within such communities, inconsistencies persist. In Ichneumonoidea alone, there are at least five different nomenclatorial systems for wing venation. As a result, communication is hindered even among experts.

Terminology is also not stable. As morphological understanding evolves, so does nomenclature. For example, in bees and apoid wasps, the “dorsal surface of the propodeum” was once called the “metapostnotum” (Brothers 1976). More recently, it has reverted to the previous name (Meira et al. 2024). Such shifts further limit accessibility and comparability across studies. Furthermore, taxonomic publications are written in many different languages, each with their own jargon making universal communication even more challenging (Tautz et al. 2003). To add to the complexity, such specialized terminology is resistant to automated translation tools.

In contrast, measures such as sequence similarity or divergence provide a standardized and quantifiable basis for comparison. Their interpretation still requires appropriate context, as the use of fixed divergence thresholds across genes or taxa can be misleading due to differences in evolutionary rates. Still, molecular data remain inherently more standardized and interoperable than morphology-based systems. DNA sequences are composed of discrete, universally comparable character states that can be stored, shared, and analyzed using common computational frameworks, independent of taxon-specific terminology. In this sense, the advantage of molecular data lies not in simplicity of interpretation, but in the standardization of representation. This is not to diminish the value of morphology. Illustrated keys and high-quality images can be highly effective for many illuminated taxa (e.g., *Megarhyssa* Ashmead (Hymenoptera), and Syrphidae (Diptera)) (Pook et al. 2016; Skevington et al. 2019). Keys can also be useful in constrained contexts within dark taxa, such as host-specific gall midges (Cecidomyiidae) (Dorchin et al. 2022).

In contrast, DNA-based identification provides a standardized and globally interoperable framework that is not constrained by terminology, language, or user expertise. This framework is already widely implemented in taxonomic research and biodiversity monitoring, enabling consistent communication and comparison across studies and regions without reliance on taxon-specific vocabularies.

### 5. Developmental inclusivity

Morphological taxonomy in dark taxa is typically restricted to a subset of life stages or sexes. For braconid wasps, it is usually adult females, since the length and shape of the ovipositor are often diagnostic. The keys to dark taxa of Diptera, e.g., Cecidomyiidae, Phoridae (Disney 1994; Brown 2010), and Tachinidae, usually deal with adult males due to the diagnostic utility of male terminalia. In these cases, and many more, it is difficult or impossible to associate the sexes without rearing or DNA barcoding. In some of the darkest groups, keys are usually incomplete, written exclusively for one sex, and immature stages are almost entirely neglected.

Importantly, some of the most consequential applications of taxonomy, ranging from pest identification in agriculture, public health, forensic entomology, and ecological research, frequently involve specimens that are immature, fragmentary, or otherwise not identifiable using traditional morphological characters. By providing a single, consistent framework for identification across developmental stages, molecular data overcome a fundamental limitation of morphology-based taxonomy and greatly expand the scope of identifiable biodiversity.

Molecular data enable identification across sexes, castes, life stages, and even from tissue fragments and environmental DNA (e.g., soil, air, water, flower surfaces). Early demonstrations of this capability include the association of life stages using DNA barcoding (Miller et al. 2005), and such approaches are now widely applied.

### 6. Comprehensive coverage

Species descriptions typically include both diagnoses and descriptions (Favret 2024). Diagnoses distinguish a new species from similar, already described taxa—usually within the same genus and geographic region—while descriptions aim to differentiate the species from all others, including those not yet described. In practice, however, this goal is difficult to achieve when only a small fraction of the fauna is known.

In hyperdiverse groups, most species remain undescribed, making it impossible to anticipate the full range of character variation needed for reliable discrimination. For example, in Neotropical *Mesochorus*, Dasch (1974) only recognized 15 species as likely to occur in Costa Rica. It would have been impossible for him to select the character state combinations that differentiate these 15 species from the other ~673 Costa Rican species that he had not encountered (Sharkey et al. 2023). Such problems are even more pronounced in megadiverse taxa like gall midges or mites which may include several million species worldwide (Hebert et al. 2016).

DNA barcode reference libraries are also incomplete and currently represent only a subset of described and undescribed biodiversity. In terms of absolute coverage, neither morphological taxonomy nor molecular databases approach completeness. However, the key distinction lies not in the proportion of species currently represented, but in how each framework accommodates unknown diversity.

DNA barcode data provide a framework that explicitly accommodates both described and undescribed diversity. Rather than requiring prior knowledge of all taxa, barcode sequences cluster specimens into discrete, closely related groups, allowing unknown specimens to be placed relative to known lineages even in the absence of formal descriptions. In morphology-based systems, unknown species must be interpreted through incomplete character frameworks, whereas molecular systems allow unknown diversity to be represented directly as discrete, comparable units.

As sequencing costs continue to decline (Hebert et al. 2018, 2025; Srivathsan et al. 2021) and additional markers are incorporated where needed, molecular frameworks will further improve resolution in cases where standard barcodes alone are insufficient (Ratnasingham and Hebert 2013).

### 7. Cryptic diversity

Morphology alone frequently fails to detect cryptic diversity, leading to the underestimation of species richness. For example, Leathers and Sharkey (2003) recognized 39 species of *Alabagrus* (Hymenoptera, Braconidae, Agathidinae) from Costa Rica in their revision which was based entirely on morphology. The *Alabagrus* fauna of Costa Rica was subsequently revised by reinforcing morphology with COI barcodes and host data (Sharkey et al. 2018). This analysis revealed that 17 of the 39 species in Leathers and Sharkey (2003) were incorrectly lumped with species that do not occur in Costa Rica. Additionally, five species from Costa Rica were found to be cryptic species complexes. Critically, Sharkey et al. (2018) could not morphologically separate three species (*A. jennyphillipsae*, *A. isidrochaconi*, *A. jeanfrancoislandry*) although these species were clearly distinguished by COI barcodes and divergent host use.

In another example, Hebert et al. (2004) used DNA barcoding, combined with host preferences and larval traits, to reveal that the skipper butterfly *Astraptes fulgerator* (Walch) is a complex of ten morphologically cryptic sympatric species (now eleven, Janzen & Hallwachs, unpublished). *Anopheles gambiae* Giles (Diptera: Culicidae) is another prime example, with at least eight morphologically indistinguishable but genetically differentiated species recognized in 2013 (Coetzee et al. 2013), though presumably this has now been resolved with molecular data. Finally, Basset et al. (2025) showed that 11.4% of insect species studied in a tropical rainforest in Panama are likely to be cryptic species. These are not isolated examples; even among vertebrates, cryptic species complexes are regularly discovered using molecular data, e.g., giraffes (Kargopoulos et al. 2024), king cobras (Das et al. 2024), and anacondas (Rivas et al. 2024).

It is important to distinguish between different forms of cryptic diversity. In some cases, so-called “cryptic species” are later resolved through more detailed morphological or ecological study, whereas in other cases species remain indistinguishable despite extensive investigation using non-molecular data. Conversely, some taxa that are clearly distinguishable morphologically show little or no divergence in standard barcode markers. Thus, no single data type is universally sufficient for delimiting all species boundaries. Integrative taxonomy remains the gold standard.

Barcode-based clustering is also not always perfectly congruent with species boundaries defined using other data types, with agreement typically in the range of 90–95% of cases (Ratnasingham and Hebert 2013). In some cases, recently diverged species or taxa with introgression may not be resolved by standard barcode markers. Despite these limitations, the key advantage of molecular data lies in their ability to detect and flag potential cryptic diversity in a consistent and scalable manner. Even when species boundaries require refinement through additional data, barcode-based clustering provides an initial, repeatable framework for identifying candidate lineages that would otherwise remain unrecognized.

These challenges highlight the importance of integrating multiple lines of evidence when refining species hypotheses. However, molecular data currently provide the only consistent means of detecting cryptic diversity at scale, allowing species-level units to be identified even when morphology offers no diagnostic signal.

### 8. Less dependence on physical access to type specimens

The comparison of new material with type specimens has long been considered best practice in taxonomy. Traditionally, this has been accomplished by either borrowing type specimens or visiting host museums. Both options are increasingly difficult to execute. Many museums no longer allow the shipment (even hand-carried) of holotypes, and the dispatch of specimens has become more complex and expensive because of the combination of national and international restrictions on the movement of biological materials, and shipping regulations related to materials that are imagined to be hazardous. Travel to collections is also costly and often unsustainable.

Even if a visit is possible, older holotypes are often in poor condition and important diagnostic features (e.g., appendages, abdomens) may be missing. Other problems are taxon specific, such as the need to dissect and examine genitalia, holotypes based on a specimen of the inappropriate sex, caste, or life stage, morphologically cryptic species, and the loss or damage of holotypes, including fire (Solly 2018). We emphasize that type specimens remain fundamental to the Linnaean system and species names are permanently anchored to them. Our argument is not for reducing their importance, but for reducing the need for direct physical access to them in routine taxonomic work.

Molecular data offer a practical pathway to reduce reliance on direct physical access to type specimens while preserving their central role in nomenclature. The recovery of DNA from type material, enabled by increasingly refined protocols for degraded or historical specimens, can provide unambiguous, comparable characters that are easily shared across research communities. Recent advances demonstrate that such data can often be obtained non-destructively or with minimal sampling (Fong et al. 2023). Where DNA preservation is poor (e.g., slide-mounted or formalin-fixed material), alternative approaches will be required, but these cases are likely to diminish as molecular data become standard in new species descriptions. By linking type specimens to molecular data, particularly DNA barcodes, their diagnostic information can be made globally accessible without repeated handling or transport. This shifts the role of type specimens from physically examined objects to digitally accessible reference points.

More broadly, molecular data are inherently more accessible than physical specimens. Sequence data can be shared globally without the logistical constraints associated with specimen loans, allowing taxonomic comparisons to be conducted remotely and reproducibly. At the same time, the long-term utility of this approach depends on maintaining open access to genetic data. Recent discussions at the international level have highlighted the importance of ensuring the continued exchange of digital sequence information for taxonomy and biodiversity research (CGRFA 2025). In this context, molecular data do not replace type specimens but extend their accessibility and utility.

### 9. Community expansion

Species identification of dark taxa is currently restricted to a small number of specialists, and even these experts often struggle to identify described species reliably. This limitation persists even in relatively well-studied faunas. For example, although Western European insects are among the best documented globally, many groups, particularly the darkest taxa like Cecidomyiidae, Phoridae, and Platygastridae, remain difficult to identify using morphology alone (Hebert et al. 2016; Ronquist et al. 2020; Hartop et al. 2024). Most species in this fauna were described prior to the widespread use of molecular data and lack sequence-based diagnostics.

One way to assess the utility of morphology-based taxonomy is to examine its congruence with DNA barcode data. To do this, we compared BIN assignments with morphology-based identifications for two large braconid genera (*Bracon* Fabricius and *Aleiodes* Wesmael) across six relatively well-sampled European countries (England, Finland, Germany, Norway, The Netherlands, and Sweden; searches conducted 15 April 2025). Both genera are globally hyperdiverse, with thousands of undescribed species. In Western Europe, most species were described using traditional morphology-based treatments that lack molecular data. Although *Aleiodes* has benefited from recent revisions incorporating biological and some molecular data (van Achterberg and Shaw 2016; van Achterberg et al. 2020), many species remain difficult to identify, and species limits may be uncertain. The situation is more severe for *Bracon*, for which no comprehensive modern treatments exist.

Because BINs are not perfect proxies for species, we also examined Finnish Geometridae, a group that has been intensively studied using both morphological and molecular approaches. Identifications on BOLD have been verified by coauthor Mutanen (Mutanen et al. 2016; Roslin et al. 2021), providing a well-supported benchmark.

We found that 301 of 305 BINs (99%) of Finnish Geometridae correspond to morphologically recognized species, with molecular data failing to distinguish species in only ~1.4% of cases (Table 1). In contrast, congruence between morphology and barcode data is much lower for *Aleiodes* (46.5%) and *Bracon* (37.9%) (Table 1). These discrepancies include both multiple species assigned to a single BIN and cases where a single species name is applied to multiple unrelated BINs (Table 1). In some instances, morphologically defined species are split across multiple BINs that form coherent clusters, suggesting that the BIN algorithm may over-partition species rather than contradict morphology. For example, 12 of 305 Finnish Geometridae species show such clustering, and similar patterns are observed in *Aleiodes* and *Bracon*.

**Table 1.** BIN statistics for Geometridae of Finland (Geos), and for the braconid genera *Aleiodes* and *Bracon* of western Europe.

**Metric**	**Geos**	** Aleiodes **	** Bracon **
Total number of BINs with >1 named specimen	305	43	58
BINs with a unique species name not shared with other BINs (non-sister, non-mono)	301 (99%)	20 (47%)	22 (38%)
BINs with more than one named specimen	294	29	25
BINs with more than one species name	5 (2%)	9 (31%)	12 (48%)
BINs with 2 species names	5 (2%)	4 (14%)	9 (36%)
BINs with 3 species names	0	2 (7%)	1 (4%)
BINs with 4 or more species names	0	3 (10%)	2 (8%)
Species in two BINs that are not sister species	0	15(52%)	11 (44%)
Species in three BINs that do not form a monophyletic group	0	4(14%)	3 (12%)
Species in four or more BINs that do not form a monophyletic group	0	1 (3%)	2 (8%)

Most of the researchers responsible for these identifications deposited their material in European museums, indicating that the issue is not a lack of expertise. Rather, it reflects the difficulty of applying morphological keys, descriptions, and images in hyperdiverse taxa. In some cases, apparent mismatches may also reflect unresolved species complexes (as in *Astraptes*), suggesting that morphology-based species concepts themselves may be incomplete.

As another example, Stribling (2006) states that for freshwater benthic invertebrates, organisms of high importance for monitoring water quality, morphologically-based identifications with error rates of 10–15% are viewed as acceptable at the genus level, but that 45% error rates do occur. Furthermore, the certainty of morphological identifications has been found to be positively correlated with taxon abundance (Stribling and Leppo 2020). In other words, the rarer a taxon is, the greater is the uncertainty associated with its identity. As a final example, a quality assessment of mosquito identifications within the MediLabSecure Network which includes laboratories in 19 countries around the Mediterranean and Black Seas found that only 64% of identifications were correct at the species level (Jourdain et al. 2018). With reliance on sequence data, scientists and even non-specialists can identify specimens with much higher confidence.

At the same time, access to molecular infrastructure remains uneven globally. In many regions of the Global South, barriers such as equipment costs, import restrictions, and limited training opportunities continue to constrain the use of DNA-based methods. As a result, the benefits of molecular identification are not yet equitably distributed, and expanding access to these tools remains a major challenge for the field.

Where accessible, molecular identification frameworks substantially lower community barriers to identification. DNA barcoding enables consistent and reproducible species identification without requiring extensive taxonomic expertise, allowing ecologists, biosecurity personnel, and non-specialists to participate with greater confidence.

### 10. Reproducibility

Reproducibility—the ability of independent researchers to arrive at the same identification or species delimitation using the same data—is a fundamental requirement of scientific taxonomy. However, morphology-based identifications are often difficult to reproduce. For example, Stribling et al. (2008) found that even after conference calls to discuss different taxonomic, morphology-based decisions a consensus could not be achieved for 14% of benthic invertebrate identifications. Such discrepancies arise because morphological characters are frequently continuous, context-dependent, and subject to interpretation.

In contrast, molecular data provide a more standardized and reproducible basis for comparison. DNA sequences consist of discrete, quantifiable character states that can be consistently generated, shared, and reanalyzed. While interpretation of these data still requires biological context, the underlying observations are directly comparable across studies and observers. This enables species hypotheses to be tested and revisited in a repeatable manner.

Reproducibility is particularly important in cases where distributions of species are discontinuous, causing the assignment of allopatric groups to populations, subspecies, or species to be somewhat arbitrary (Mutanen et al. 2012; Joshi et al. 2024). Species delimitation based on DNA characteristics enables repeatable, standard means to characterize species limits. Under circumstances where species delimitation is inherently difficult, taxonomic stability is best served if species are defined based on repeatable, standardized criteria. Morphological traits are often continuous in nature, making morphological delimitations difficult to replicate, providing little basis for taxonomic stability under such circumstances.

At the same time, integrative approaches remain important for refining species hypotheses. Morphological data can provide critical validation for MOTUs (molecular operational taxonomic units) such as COI BINs (Hartop et al. 2022). For example, the treatment of Costa Rican Braconidae by Sharkey et al. (2021) found that ten species of *Macrocentrus* (Hymenoptera: Braconidae) were nested in the same BIN (BOLD:ACK7466). However, the members of each of these species formed a discrete sequence cluster so they are diagnosable with barcode data. In short, the molecular data do not conflict with the morphological results, but rather the species are so young that their divergences are too low to lead to their assignment to different BINs. The first clue that these clusters represented distinct species was their possession of diagnostic morphological traits which allowed Sharkey et al. (2021) to construct a morphological key to diagnose them. The second clue was that each cluster had a unique set of host caterpillars. Without integrative morphological and host data, barcodes may have failed to recognize these species.

Similar complexities occur in other systems. Some specimens of two Finnish geometrids, *Scopula frigidaria* (Möschler) and *Scopula ternata* Schrank, share identical barcodes. In this case the two species are distinguishable morphologically, but where their ranges overlap the two are hypothesized to have mitochondrial introgression because barcode sharing occurs only in proximity to areas where the species co-occur (Hausmann et al. 2013). In other cases, such as sawflies where COI performance is reduced by the prevalence of NUMTS, nuclear markers can improve resolution (Prous et al. 2025).

These examples do not undermine the reproducibility of molecular data but rather highlight the need for integrative approaches when refining species limits. Molecular data provide a consistent and repeatable foundation upon which additional data types can be layered. In contrast, morphology alone often lacks this baseline reproducibility.

### 11. Taxonomic stability

DNA barcoding has revealed a vast amount of previously unrecognized diversity over a relatively short time frame, with more than 1.4 million BINs generated in the past two decades, compared to **~**1.57 million described species accumulated over more than two centuries (BOLD Systems; accessed 4 Jan 2026). For many dark taxa, there are already far more BINs than described taxa. For example, there are ~23,000 described species of Braconidae worldwide (Yu et al. 2016), but there are currently (20 Aug 2025) 32,988 braconid BINs on BOLD, a number that will increase substantially as geographic sampling improves. This disparity is illustrated by regional differences: Brazil currently has only 184 BINs of Braconidae, while tiny Costa Rica has 8,562 braconid BINs (23 May 2025) with samples from only 3% of its land area (DJ and WH, unpublished data).

The gap between described and actual diversity is even more pronounced in other dark taxa. In gall midges (Cecidomyiidae), only 100 named species of this family were documented in Canada as of 2016, yet barcode data suggest that there are more than 20,000 species in the country (Hebert et al. 2016). If this represents ~1% of global gall midge species diversity (thought to be Canada’s share), there could be 2 million species of cecidomyiids worldwide. The currently described species cover only 0.34% of this diversity. Even for relatively well-known taxa such as bees of the family Apidae there are 37% more BINs from Thailand on BOLD (170) (3 Feb 2025) than there are species recorded (124) from that country (Packer, unpublished). Moreover, as sequences accumulate on BOLD, species distributions will become better understood and more readily accessible and informative than those in the literature. If taxonomists wish their newly described species to be included in the broader context of global ecological and geographic distributions, it is essential that DNA barcodes be recovered from them.

As barcode data accumulate, they provide increasingly accurate and accessible information on species boundaries and distributions, often surpassing what is available in the literature. However, taxonomic stability is not achieved through molecular clustering alone. Stability emerges when Linnaean names are consistently linked to molecular data, particularly through sequencing of type specimens or topotypic material (Sánchez-C et al. 2022; Renner et al. 2024). In this way, species names become anchored to standardized and reproducible reference points that can be reliably applied across studies, regions, and time. To retain and strengthen the utility of the Linnaean system, it is therefore essential that new species descriptions include DNA barcodes and that existing names are progressively connected to molecular reference data. Without this linkage, both molecular and traditional taxonomic frameworks remain incomplete.

BINs are not static entities; their composition can change as new data are added and clustering algorithms are refined. As such, they are not a substitute for formally described species, but rather a dynamic framework for organizing and exploring biodiversity.

### 12. Future relevance

For dark taxa, species identifications will increasingly rely on molecular sequence data, and taxa lacking such data risk becoming effectively invisible in emerging areas of biodiversity research. A growing number of technologies that are transforming our understanding of biodiversity interactions and species distributions depend directly on molecular data, and their benefits are largely restricted to taxa with sequence-based diagnostics.

One such development is the use of high-throughput sequencing to recover entire symbiomes from individual specimens. Deep sequencing of a specimen can recover symbiomes, either as low-abundance variants obtained with the barcode primers themselves, or using taxon-specific primers that avoid co-amplification of the ‘host’ DNA. For example, when an entire insect is barcoded, so too will be its prey (Pons 2006) and any parasitoids (Sow et al. 2019). Using additional primers, e.g., targeting food plants, associated fungi, and bacteria can recover entire symbiomes (Grant et al. 2021). If the insect and its component symbiome have Linnaean names that are not barcoded, the ecological information will rest solely with the constituent sequences. Given enough time, the BIN system or its successor will be far more informative than currently published literature for food networks and species interactions in general.

A second major advance is metabarcoding, in which bulk samples (e.g., Malaise trap collections) are sequenced simultaneously to characterize biodiversity (Liu et al. 2020). This technology can be used to determine the distributional ranges of organisms, access local biodiversity (Thomsen and Sigsgaard 2019), endemism, and change over time for environmental assessment, inexpensively and rapidly. However, the application of these and other advances like environmental DNA analysis are restricted to species that have been registered in a DNA barcode reference library.

Together, these developments highlight a fundamental shift in biodiversity science: species without molecular data will be excluded from many of the most powerful tools for studying ecology and distribution. Incorporating DNA barcodes into species descriptions is therefore essential to ensure that newly described taxa remain accessible and relevant in an increasingly sequence-based research landscape. We do not currently have an equivalent pathway for linking species to these rapidly expanding sources of ecological and distributional data with other forms of data. Any such alternative system would require substantial time and development to reach the level of integration already achieved by DNA barcoding.

## Discussion

### Access, equity, and feasibility of molecular taxonomy

DNA barcoding has become increasingly affordable, enabling decentralized sequencing and supporting the vision of “identification by everyone, for everyone” (Srivathsan et al. 2021). However, affordability remains uneven. Even modest sequencing costs may remain beyond the reach of many institutions in biodiversity-rich regions of the Global South. Nevertheless, when compared to the costs of traditional taxonomy, particularly for dark taxa, molecular approaches may be more accessible and are certainly more scalable.

Morphology-based identifications for many invertebrates, such as parasitoid wasps or certain Diptera, typically require direct comparison with type specimens, the majority of which are curated in institutions located in Europe or North America. This entails lengthy international loans or, more commonly, overseas travel for specialized taxonomists, incurring costs for visas, flights, accommodation, per diems, and insurance, totaling several thousand USD for a single study trip. In contrast, portable sequencing platforms such as ONT's MinION, enable local processing of large sample sets within days. For example, a MinION starter pack (1,999 USD, 22 Aug 2025) and a series of Flongle flow cells can yield thousands of DNA barcodes, allowing in-country researchers to delimit species without the logistical and financial burden of accessing foreign collections (Hebert et al. 2025). In Ecuador’s Chocó region, the cost of one mobile sequencing setup was less than the combined travel and lodging expenses of sending two researchers to a European museum for a two-week type specimen study and, crucially, the molecular data can be generated and analyzed in-country (Diego J. Inclán, unpublished).

For dark invertebrate taxa, for which diagnostic morphological characters are often subtle, highly variable, or dependent on male genitalia preparations, molecular data can quickly triage operational taxonomic units, flag potential new species, and enable targeted morphological study. This approach reduces the need for expensive visits to foreign museum visits, and channels funds toward building local capacity and collaborative verification, making taxonomy more feasible and sustainable for institutions in the Global South. Taxonomic research in the Global South would be greatly facilitated if holotypes housed in former colonial countries were DNA barcoded. The authors believe this should be a fiscal priority for such institutions. Infrastructure support, open training resources, and equitable international collaborations are critical to democratize access to molecular tools. Recent initiatives, such as the Genomics of the Brazilian Biodiversity, show that it is possible to overcome most of the limitations with concerted efforts (Vilaça et al. 2024).

Still, not all countries in the Global South are as fortunate as Ecuador and Brazil. For many, the importation of scientific equipment is complicated by high import taxes, inefficient logistics, and lengthy bureaucratic procedures, all of which increase costs. Additionally, there is a persistent shortage of trained professionals and high staff turnover, largely due to unstable employment conditions. These barriers are a common issue across many developing and transition countries, contributing to scientific capacity gaps among nations.

Therefore, it is important to recognize that morphology-based approaches continue to play a critical role, particularly in regions where molecular infrastructure is limited. Species can and will continue to be described without DNA data. However, such approaches often face constraints in scalability, reproducibility, and accessibility, particularly for dark taxa. Incorporating molecular data whenever possible therefore represents not a replacement for existing practices, but a means of extending their impact and long-term utility.

### Emerging approaches

Importantly, our argument is not that molecular data are intrinsically superior to other forms of evidence, but rather that they currently provide the only globally standardized framework that satisfies all 12 operational requirements discussed in this paper simultaneously. We fully expect imaging, automation, robotics, and artificial intelligence to play an increasingly important role in taxonomy and biodiversity science, and we view these developments as highly complementary to molecular approaches rather than as competing alternatives. High-throughput imaging systems are advancing rapidly, and large-scale initiatives are now generating standardized image datasets that demonstrate the considerable promise of machine learning for specimen identification, automated sorting, and biodiversity monitoring (e.g., Steinke et al. 2024). We are actively involved in these developments and regard them as an important component of the future taxonomic toolkit.

Nevertheless, despite impressive recent progress, image-based approaches do not yet provide a broadly applicable framework capable of meeting the practical requirements of large-scale taxonomy at the same level as molecular approaches. Their performance depends on extensive, curated training datasets and remains sensitive to imaging conditions, specimen preparation, and taxonomic coverage. While machine-learning systems can achieve remarkable accuracy for well-characterized groups, they currently struggle to accommodate the vast amounts of undescribed diversity that characterize dark taxa. This challenge is particularly acute because dark taxa are typically composed of small-bodied organisms whose diagnostic features are often subtle, variable, or inaccessible to routine imaging. In many groups, critical characters require dissection, slide mounting, scanning electron microscopy, or increasingly sophisticated three-dimensional imaging techniques such as micro-CT or synchrotron tomography. As a result, even generating the image data needed for species delimitation can be labor-intensive and difficult to standardize across taxa and studies. In many dark taxa, the challenge is not simply recognizing species from images but determining which structures must be imaged. Unlike DNA sequences, images do not yet provide a universally standardized and interoperable data layer that can be readily compared across projects, institutions, and geographic regions. Furthermore, image-based approaches remain fundamentally limited in their ability to detect cryptic diversity, represent unknown species as stable and comparable units, or incorporate the wide range of biological material accessible through molecular methods, including immature stages, tissue fragments, environmental samples, and environmental DNA.

These limitations are not necessarily permanent. Continued advances in imaging technologies, specimen digitization, artificial intelligence, and data infrastructure may eventually yield frameworks that rival the scalability and utility of molecular approaches. Indeed, the future of taxonomy will likely involve the integration of molecular, morphological, imaging, ecological, and behavioral data in increasingly automated workflows. However, no globally adopted equivalent of a DNA barcode reference library currently exists for image-based taxonomy, nor is there an image-based framework that provides comparable interoperability across biodiversity research, environmental monitoring, metabarcoding, eDNA studies, and other rapidly expanding areas of biology. Until such systems achieve comparable levels of standardization, transferability, and operational maturity, molecular data remain the most effective and broadly applicable foundation for scalable taxonomy.

### Policy, infrastructure, and the future of taxonomy

Beyond economics, regulatory and policy barriers threaten progress. Restrictive permitting regimes and debates surrounding digital sequence information are curbing access to the sequence data that underpin modern taxonomy. The risk of barriers to the sharing of digital sequence information at both national and international levels is a concern. The Commission on Genetic Resources for Food and Agriculture, an international body, recently recognized this challenge within the context of regulations on access and benefit-sharing, noting the “need for open exchange of genetic sequence information and biomaterials required for specimen identification” (CGRFA 2025: paragraph 83). An international framework is urgently needed to safeguard unrestricted access to the short diagnostic sequences critical for species discovery, conservation, and ecological monitoring.

The potential of molecular tools is already being realized at scale. Since 2023, the Centre for Biodiversity Genomics at the University of Guelph has barcoded three million specimens annually for ~$2 per sample, work which has added 150,000 BINs each year. This effort highlights the transformative power of sequencing but also reveals current limitations. While BINs as operational taxonomic units are invaluable proxies for species, their utility would be greatly augmented by features such as persistent, time-stamped records and trackable membership histories, ensuring transparency and reproducibility.

The world is currently losing species at a faster rate than we describe them (Costello et al. 2013; Woinarski et al. 2024). Therefore, the use of DNA barcoding and derived technologies (eDNA, metabarcoding) are critical as they have the potential to enable conservation action, even for species that lack a Linnean name.

## Conclusions

Because all data types (morphological, behavioral, ecological, and molecular) are valuable, integrative taxonomy remains the gold standard because it draws strength from multiple lines of evidence. As outlined in this paper, a modern taxonomic framework must meet a set of practical requirements, including scalability, universality, reproducibility, and applicability across life stages and unknown diversity. At present, DNA barcoding is the only data type that consistently satisfies these criteria in a standardized and operational way. Emerging technologies that combine robotics with advanced imaging and analytical approaches will likely reshape the taxonomic landscape in the future, but a standardized, globally applicable framework for their routine use is not yet in sight.

Therefore, for now, for dark taxa, molecular data are not a luxury; they are essential. Hyperdiverse taxa cannot be meaningfully studied or described with morphology alone. Without molecular data, these species remain invisible, misidentified, or excluded from scientific and conservation agendas.

This paper is a call to action. The tools now exist to illuminate the darkest corners of biodiversity. Sequencing is affordable, scalable, and accessible. What remains is the collective will to embrace these tools across disciplines, institutions, and borders. To keep pace with the biodiversity crisis, taxonomy must replace outdated conventions with open, equitable, and reproducible practices that empower the global bioscience research community.

For taxonomy to remain relevant, it must be built on foundations that reflect biological realities. That means investing in molecular infrastructure, reforming publication standards, and ensuring that none of the species shaping our ecosystems, however small or poorly known, are left behind.

## Additional information

### Artificial Intelligence (AI) use

The authors accept full responsibility for the content of the manuscript, including the disclosure of any use of AI.

No AI tools were used in the preparation of this manuscript.

### Author ORCIDs

M.J. Sharkey https://orcid.org/0000-0001-6201-7340

M. Mutanen https://orcid.org/0000-0003-4464-6308

B.V. Brown https://orcid.org/0000-0001-6367-6057

B.A. Butcher https://orcid.org/0000-0002-0541-0709

H.C.J. Godfray https://orcid.org/0000-0001-8859-7232

W. Hallwachs https://orcid.org/0000-0002-5166-809X

A. Hausmann https://orcid.org/0000-0002-0358-9928

P.D.N. Hebert https://orcid.org/0000-0002-3081-6700

D.J. Inclán https://orcid.org/0000-0001-5323-3674

D.H. Janzen https://orcid.org/0000-0002-7335-5107

N. Kiljunen https://orcid.org/0000-0001-6397-3254

S.E. Miller https://orcid.org/0000-0002-4138-1378

G. Oliveira https://orcid.org/0000-0003-0054-3438

L. Packer https://orcid.org/0000-0002-1711-8203

J.H. Skevington https://orcid.org/0000-0002-1445-9870

D. Steinke https://orcid.org/0000-0002-8992-575X

A.P. Vogler https://orcid.org/0000-0002-2462-3718

E. Hartop https://orcid.org/0000-0002-8598-9844

### Data availability

All of the data that support the findings of this study are available in the main text.
